# Motor Contagion during Human-Human and Human-Robot Interaction

**DOI:** 10.1371/journal.pone.0106172

**Published:** 2014-08-25

**Authors:** Ambra Bisio, Alessandra Sciutti, Francesco Nori, Giorgio Metta, Luciano Fadiga, Giulio Sandini, Thierry Pozzo

**Affiliations:** 1 Department of Robotics, Brain and Cognitive Sciences, Istituto Italiano di Tecnologia, Genoa, Italy; 2 iCub Unit, Istituto Italiano di Tecnologia, Genoa, Italy; 3 Dipartimento di Scienze Biomediche e Terapie Avanzate-Sezione di Fisiologia Umana, Università degli Studi di Ferrara, Ferrara, Italy; 4 INSERM, U1093, Cognition Action Plasticité Sensorimotrice, Dijon, France; 5 Institut Universitaire de France, Université de Bourgogne, Campus Universitaire, UFR STAPS Dijon, France; University of Bologna, Italy

## Abstract

Motor resonance mechanisms are known to affect humans' ability to interact with others, yielding the kind of “mutual understanding” that is the basis of social interaction. However, it remains unclear how the partner's action features combine or compete to promote or prevent motor resonance during interaction. To clarify this point, the present study tested whether and how the nature of the visual stimulus and the properties of the observed actions influence observer's motor response, being motor contagion one of the behavioral manifestations of motor resonance. Participants observed a humanoid robot and a human agent move their hands into a pre-specified final position or put an object into a container at various velocities. Their movements, both in the object- and non-object- directed conditions, were characterized by either a smooth/curvilinear or a jerky/segmented trajectory. These trajectories were covered with biological or non-biological kinematics (the latter only by the humanoid robot). After action observation, participants were requested to either reach the indicated final position or to transport a similar object into another container. Results showed that motor contagion appeared for both the interactive partner except when the humanoid robot violated the biological laws of motion. These findings suggest that the observer may transiently match his/her own motor repertoire to that of the observed agent. This matching might mediate the activation of motor resonance, and modulate the spontaneity and the pleasantness of the interaction, whatever the nature of the communication partner.

## Introduction

It is well known that movement observation has measurable effects on the observer's motor system, and that these are attributable to the activation of mirror neuron circuits [1]. These brain areas give rise to a series of “resonance behaviors” in which, during the observation of actions performed by others, motor representation congruent with the observed actions becomes automatically activated in the observers' brain [2]. In such a context, if the motor system is prepared to produce a motor response to the observed motion, this might result in motor contagion: namely, the observer's motor performance might automatically replicate some features of the stimulus. Since motor resonance was proposed to affect humans' ability to interact with others, yielding the kind of “mutual understanding” at the basis of social interaction [3], [4], several behavioral, neurophysiological and neuroimaging studies have dealt with this matter. However, it remains still unclear how different action features combine/compete to promote or prevent motor resonance, and specifically motor contagion (for a review see [5]).

Indeed, when observing a moving agent there are several sources of information that can influence the observer's motor response (for a review on bottom-up and top-down effects see [6]). The *nature of the observed agent* in its physical appearance might affect on the way people react to his/her actions. For instance, whether a mechanical device (e.g., a robot) is able to evoke motor resonance has been a source of debate. Early studies on the mirror neurons system in the monkey [7], [8] and behavioral [9] and neuroimaging findings in humans [10] suggest that only biological stimuli evoke motor resonance. However, recent neuroimaging, neurophysiological and behavioral experiments contradicted these results by showing that the observation of human and robotic movements induced comparable neural activations [11], [12] and behavioral motor responses [13]. A more subtle explanation going beyond the simple physical appearance of the agent was tested by a series of studies that compared the interference effect measured during the interaction with either a human agent or a robotic arm. These works suggested that motor priming is modulated by the attribution of a social intention to the interactive agent: an attribution that is feasible in the case of a human partner but is precluded when interacting with a robot [14], [15]. However, the robot employed in these experiments was a simple robotic hand wearing a glove and mounted on a metal frame. Thus the stimulus was perhaps not sufficiently humanoid to trigger motor resonance and consequent modulation of motor behavior.

There are other elements of an action that might play a role in making interaction smooth or awkward. Indeed, actions can be described on at least two levels: by considering their meaning (the goal of the action) or their kinematic properties (the features of the movement itself).

The action meaning can be seen as a high level property, which may vary according to the presence or the absence of an object during motion – *object-directedness*. Behavioral studies on humans showed that when the observed action was goal oriented, participants naturally focused on and imitated its goal, rather than other action features such as movement kinematics [16]. Indeed, it seems that the presence or absence of objects as goals of the movement has a decisive influence on imitation behavior [17]. In particular, goal-oriented movements seem to be imitated correctly with respect to the goal, but the movement itself is frequently ignored [18], [19]. However, other studies contradicted these results by showing that even in the presence of goal objects, participants focused on the motor components of the actions [20]–[22]. Furthermore, daily-life activities often imply actions that have to be considered for both their goal and motion components. For instance, when a person gives an object to another person, the latter has to focus on the object (i.e., the goal), but also on the temporal components of the giver's movement (i.e., kinematics), so as to be ready to receive the object. Therefore, disregarding the kinematic features of an action in favor of its goal may not always be optimal. Nevertheless, no agreement was reached on the relative contributions of movement goal and kinematics when reacting to an observed movement.

Moving to a low-level action representation, movement *kinematics*, such as the velocity profile and the trajectory of the limb, might also vary the degree of motor resonance evoked in the observer. Previous researches have shown that an impoverished visual display of motion (i.e., a single dot) was sufficient to help participants to infer the missing part of an observed trajectory [23] and to evoke motor contagion in the observers' motor response [24], but only when it moved according to the biological laws of motion. A functional magnetic resonance imaging (fMRI) study failed to find increased metabolic response in the conventional mirror neuron areas during the observation or imitation of finger movements as opposed to a dot moving with the same velocity profile [25]. This result suggests that either the paradigm was not the most appropriate to unveil the differences between the two stimuli (as suggested by the authors) or that the biological velocity profile was sufficient to elicit the same neural activity as the moving finger. Conversely, the efficacy of a biologically plausible velocity profile to evoke motor resonance with a robotic device was supported by neuroimaging investigations showing that non-living agents, such as industrial robots, moving with an artificial constant velocity evoke the same neural response generated by natural human movement observation [11]. Although an interesting and detailed review suggested that a biological origin of the observed agent is a crucial factor to activate the action-observation network [26], the need of the observer to recognize his/her motor repertoire in the observed visual model still needs to be clarified.

A further low-level action property is represented by movement *shape*, i.e., the geometrical shape of the trajectory of the effector. Human actions are characterized by smooth, curved shapes, while robotic motion is typically thought of as jerky and squared. How much the trajectory shape should correspond to the nature of the moving demonstrator to evoke motor resonance in the observer is poorly documented. To the best of our knowledge only one study tested this factor by means of neuroimaging techniques, showing that the action-observation network is substantially activated by the observation of artificial movement shape, irrespective of the nature of the agent [27].

The goal of the present study was to investigate how the nature of the observed agent, the object-directedness, and the kinematic properties of the observed movement influence a human observer when interacting with an external agent. To this aim a humanoid robot and a human agent were monitored by the participant while performing transitive or intransitive actions, characterized by either a smooth-curvilinear or a jerky-artificial trajectory, and following biological kinematics. Furthermore, by using a humanoid robot, we could include an additional condition in which the robot moved with a non-biological velocity profile. Thus, we were able to test the role of correspondence between the internal motor repertoire of the observer and the one exhibited by the demonstrator. Participants were requested either to move their hands into a pre-specified final position or to put an object into a container after they had seen the demonstrator performing the same task at various execution velocities. We measured the degree of motor contagion (i.e., how much participants' velocity was affected by demonstrator' velocity), considering that the more motor resonance is evoked, the more participants' motor response is influenced by the observed motion [24], [28]. If the activation of the motor resonance mechanisms relies mostly on the possibility to match the observer's motor repertoire with that of the visual model, motor contagion will appear in all conditions, irrespective of the nature of the demonstrator (human/robot) and the shape of the covered trajectory (smooth-curvilinear/jerky-artificial), but will be absent when the demonstrator exhibits non-biological kinematics. Furthermore, if the presence of an object as action goal induces participants to ignore movement kinematics, then participants' velocities will not be modulated by the demonstrator's velocity during the observation of transitive (i.e., object-directed) actions.

## Materials and Methods

### Participants

A total of 28 healthy young adults (10 women, age 24.4±6, mean±SD) took part in the experiments. The population was split into two groups (14 participants per group, randomly assigned): one group observed the human demonstrator (H; 6 women, mean age ±SD  = 22.7±2.4), whereas the other group observed movements performed by a humanoid robot (R; 4 women, mean age ±SD  = 26.3±7.9). Since one participant of the H group did not perform all the experimental conditions, her data were not included in the analysis. Consequently, the H group was composed of 13 participants. An independent *t-test* on the age of the two groups did not find any significant difference. All participants were right-handed according to an informal interview, and had normal or corrected-to-normal vision. Written informed consent was obtained from each participant in the study, which was approved by the local ethics committee ASL-3 (“Azienda Sanitaria Locale”, local health unit), Genoa, and was in agreement with the Helsinki Declaration of 1975, as revised in 1983. The individual in this manuscript has given written informed consent (as outlined in PLOS consent form) to publish these pictures.

### Apparatus

The experiment took place in a large room. The participant and the demonstrator (robot or human) were oriented toward a wall to avoid possible disturbance that might occur during the study. Two blue cardboards squares (area: 4 cm^2^) were placed about 5 cm from the border of a table to indicate the starting movement position of the demonstrator and the participant. The table was covered in black fabric. Two blue cardboards circles, 13 cm in diameter, indicated participant's and demonstrator's motion end positions. The need to precisely reach the final position was not emphasized to prevent movement final adjustments. The distance between the centres of the square and the circle was fixed at 20 cm. This distance was determined by the motor constraints of the robot in the sagittal plane, since 20 cm represented the maximum length the robot could cover with very limited trunk displacement.

**Figure 1:**
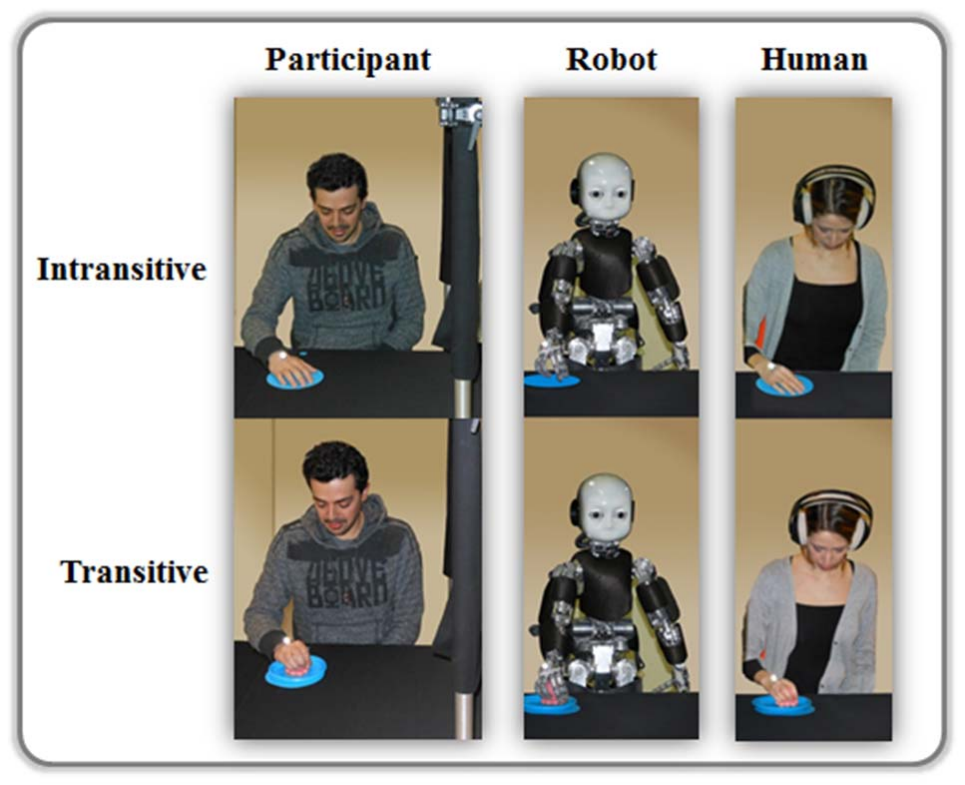
Experimental set up. Participant – on the left side of the black curtain – observed intransitive (upper row) and transitive (lower row) movements performed by the humanoid robot (left column) and by the human demonstrator (right column) –on the right side of the black curtain. In the intransitive condition the model and the participant simply moved the hand from the start to the end position indicated by a blue square and a blue circles, respectively. In the transitive movements the model and the participant moved a pink octopus from the blue square into a final blue toy fishpond. The distance between the start and the final positions was constant.

Participants were seated on a chair at the table in a comfortable position. They were sufficiently close to the table to move their arm from their start to their end position in an unrestrained manner. To guarantee that participants paid attention only to demonstrator's arm kinematics and not to other cues, such as gaze direction, a black curtain was placed between participant and demonstrator. Curtain height was adjusted for each participant so that he/she could see only the right arm of the demonstrator, while the face was hidden from view (Figure 1). Participants were seated on the right side of the demonstrator. This position was selected to allow the subject to appreciate the kinematic features of the reaching movement (especially the shape and the velocity profile) that evolved mostly in the sagittal plane.

A VICON Motion Capture System with six infrared cameras fixed on appropriate steel structure mounted on the wall (minimum and maximum linear distances from the table: 2 and 3.5 m) was used to record the demonstrator's and participant's movements at a sampling frequency of 100 Hz. The demonstrator's and participant's hands were kept in a palm down position throughout the experiment. One passive infrared reflective marker (diameter 9.5 mm) was applied to the back of the right hand of the participant and the demonstrator (Figure 1). In particular, the marker was positioned between the first and the second metacarpal bones, just above the metacarpophalangeal joint. This location was chosen by taking into account the robot structural features.

### Experimental paradigm

**Figure 2:**
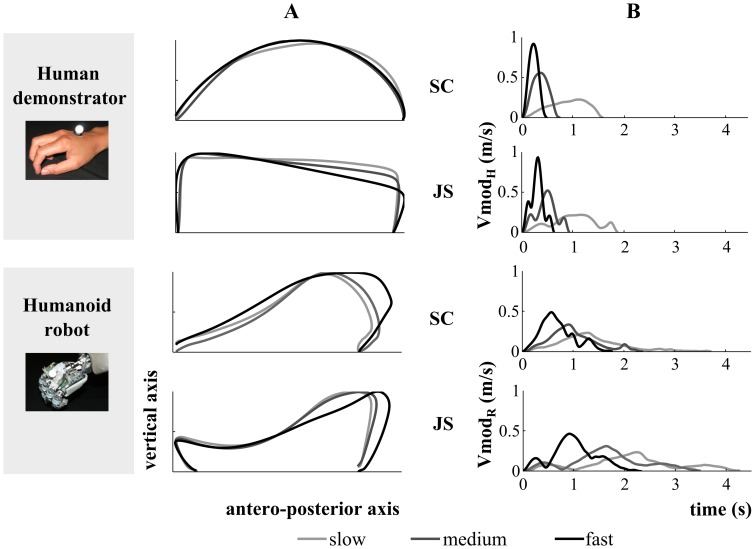
Examples of trajectories and velocity profiles when the demonstrators performed a biological movement. On the left, are represented the human and the humanoid demonstrators' trajectories in the sagittal plane; on the right, the modules of the velocity profile of the two stimuli. SC and JS refer to smooth-curvilinear and jerky-segmented trajectories, respectively. The levels of grey code movement velocities.

To consider whether the *nature of the agent* is a crucial factor to evoke motor contagion, participants observed a human agent and a humanoid robot during the execution of reaching movements performed with the right upper limb (Figure 1). The two demonstrators covered both smooth-curvilinear (SC) and jerky-segmented (JS) trajectories to test the role of motion *shape* (Figure 2). Additionally, in order to assess whether and how the presence of an *object* as goal for the reaching task influences motor resonance, the previously described movements were either intransitive (I) or transitive (T). In the Intransitive condition both the human demonstrator and the humanoid robot performed a hand displacement from the start to the end position, while in the Transitive task they moved a plastic octopus from the start position into a toy fishpond. Finally, the role of the observed movement *kinematics* in motion contagion was tested by letting the humanoid robot move according to biological (B) or non-biological (NB) laws of motion (see paragraph Stimuli for more details). Since it is not possible for a human agent to violate the biological laws of motion, the NB condition was tested only for the humanoid robot.

The participant was requested “to observe the demonstrator's movement and, when it/she stopped, to move the hand into the blue circle in front of her/him” in Intransitive condition. In the Transitive condition two plastic octopuses were placed over the starting squares and two blue plastic plates covered the final circles in front of both the demonstrator and the participant. Here the participant was requested “to observe the demonstrator's movement and, when it/she stopped, to move the octopus into the fishpond in front of her/him”. Participant's reaching movements ought to be one shot (i.e., without final adjustments or intermediate stops) and mainly occurred in the sagittal plane, as those of the two visual models. Each experimental condition was run in separate blocks and in each of them the stimulus moved at three different velocities: Slow, Medium and Fast (S, M, F).

Trajectory (SC, JS), Object-Directedness (I, T) and Velocity (S, M, F) were considered as sources of variability, resulting in a total of 120 trials (10 replications per each velocity) for each stimulus (Group: H, R). Since the R group performed an additional condition, namely the observation of non-biological movement kinematics over a curvilinear trajectory with three velocities (30 movements, for a total of 150 trials), Kinematics (B, NB) was a further source of variability only for R group.

Participants paused for at least one minute after a block of 30 trials, or as desired. The experimenter reminded participants of the instructions after each pause. The order of execution of the experimental conditions was counterbalanced in both groups, and inside each condition the 10 repetitions of the stimulus velocities occurred randomly.

### Stimuli

During the observation of the human demonstrator the person who acted as model was a woman and was the same in all the experiments. She was previously trained to make movements at three different velocities. In order to reduce variability in her movement duration, she wore a headphone connected to a PC on which a MATLAB code generated a rhythmic “beep”. This informed her on the movement duration she had to produce in each trial.

The robot used as a stimulus in this work was iCub [29], a humanoid model developed as part of the EU project RobotCub. It is approximately 1m tall with the appearance of a 3.5 year old child. To produce robot biological hand movements (B), we recorded the human demonstrator's reaching movements while covering both smooth-curvilinear and jerky-segmented trajectories at three different velocities. To design the non-biological (NB) robot movements we started from B movements and we made the velocity profile reach a maximum after a very brief initial acceleration phase, and remain approximately constant for a minimum of 1 s, before rapidly decelerating. The non-biological motion was intransitive (I) and characterized by a curvilinear shape (SC). It is worth noting that if the jerky-segmented trajectory was an unusual, but human-feasible movement, in the non-biological condition the robot violated the biological laws of motion, though covering a smooth-curvilinear path.

To evaluate the possible differences among the movements of the two demonstrators in different conditions [30], which could in turn affect participants' motor response, we statistically compared the robot and human demonstrator's total movement duration, mean and maximum velocities, minimum and maximum accelerations, length and maximum height of the trajectory. The latter parameters refer to spatial components of the movement, whereas the previous ones provide a temporal description of the motions of the demonstrators. ANOVAs with Trajectory (2 levels, SC and JS), Object-Directedness (2 levels, I and T), and Velocity (3 levels, S, M and F) as within-subjects factors, and Group (2 levels, R and H) as between-subjects factor were applied on these kinematic parameters. Statistically significant differences were found between the two demonstrators in all the mentioned parameters.

The mean values of these parameters over the 10 repetitions in each experimental condition (2 Trajectory ×2 Meaning ×3 Velocities for participants of the two Groups –13 for H and 14 for iC) were submitted to a principal component analysis (PCA) to deeply investigate the source of differences between the two demonstrators. Results indicated that the first two components accounted for the 90% (72% and 18%, respectively) of the variance. The coefficients for the first two components are reported in the Table 1.

**Table 1:** Coefficients of the kinematic parameters for the first two components.

	Component 1	Component 2
**Movement duration**	−0.39	−0.04
**Mean velocity**	0.43	−0.14
**Max velocity**	0.42	−0.25
**Max acceleration**	0.43	−0.16
**Min acceleration**	−0.43	0.15
**Trajectory length**	−0.15	−0.76
**Trajectory height**	−0.29	−0.54

The first component had a positive coefficient (≥.30) for mean and maximum velocities and maximum acceleration, whereas a negative component for movement duration and minimum acceleration. The path length and the trajectory height weighted substantially on the second component. These results suggested that the first component was strongly connected to the temporal features of demonstrators' movements, whereas the second component was related to its spatial features. We performed univariate ANOVAs to compare the two types of stimuli with respect to the two kinematic components. The effect of the stimulus type was significant for both components (Component 1: F(1,322) = 301.73, p<<0.01; Component 2: F(1,322) = 47.69, p<<0.01).

### Data treatment

#### Kinematic analysis

Kinematic data were low-pass filtered at 5 Hz using a 2nd order Butterworth filter. To define the onset and end of the movement, we chose a threshold corresponding to 2% of the maximum value of the movement velocity profile. The same processing method was applied to analyse the movements of the participants and the stimuli.

To quantify the occurrence of motor contagion we used the same procedure already applied in our previous studies [24], [28] and we considered movement mean velocity (V) as outcome parameter. Thus, we tested if the velocity of the visual model influenced participants' velocity. In each experimental condition participants and models' V were obtained by averaging mean movement velocity over the 10 repetitions.

#### Statistical analysis

The statistical analysis on demonstrators' mean movement velocities revealed that the interactions Group*Object-Directedness*Velocity (F_2,50_ = 4.4, p<0.05) and Group*Trajectory*Velocity (F_2,50_ = 53.61, p<0.01) were statistically significant. For the complete results of the statistical analysis please see Table 2. To summarize the results of the Post-Hoc comparisons applied to the previously significant interactions, we will report hereafter only the data that were useful to plan the statistical analysis of participants' performance. Smooth-curvilinear movement trajectories were always faster than jerky-segmented trajectories (V_SC_>V_JR_, p<<0.01) for both agents. Furthermore, the human demonstrator's mean velocities were always significantly higher than the robot velocities for both trajectories (V_H_>V_R_, p<<0.01) at each speed level (S, M and F). Therefore, to account for the differences in stimulus velocities, participants' motor responses were classified on the basis of Group (R and H) and Trajectory (SC and JS), for a total of four repeated-measures ANOVAs (R-SC, R-JS and H-SC, H-JS) with Object-Directedness and Velocity as factors.

**Table 2:** Results of the statistical analyses comparing human and robotic demonstrators' movement velocities.

Mixed-design ANOVA on demonstrators' V	Newman-Keuls post hoc
	R-I < H-I for S, M and F, *p<0.001*
Velocity*Object-Directedness*Group	R-T < H-T for S, M and F, *p<0.01*
*F_2,50_ = 4.4, p<0.05*	H-T < H-I for S, M and F, *p<0.001*
	R-T and R-I for S, M and F, *Not significant*
Group*Trajectory*Velocity	R-SC < H-SC for S, M and F, *p<0.001*
*F_2,50_ = 53.61, p<0.01*	R-JS < H-JS for S, M and F, *p<0.001*

The results of the mixed-design ANOVA on human (H) and robotic (R) demonstrators' mean movement velocities. On the left the interactions among the within-subject factors Velocity, Trajectory and Object-Directedness and the between-subject factor Group. On the right the result of the Newman-Keuls post-hoc comparisons focused on the differences between human and robotic movements performing transitive (T) and intransitive (I) motions, while covering smooth-curvilinear (SC) and jerky-segmented (JS) trajectories at different velocities (Slow, Medium and Fast).

Four repeated-measures ANOVAs – with Object-Directedness (2 levels, I and T) and Velocity (3 levels, S, M and F) as within-subjects factors – were applied on participants' mean movement velocity to assess whether motor contagion appeared when observing the robotic and the human demonstrator performing smooth-curvilinear and jerky-segmented trajectories.

Moreover, since in the case of motor contagion demonstrator's and participant's velocities varied coherently, for each participant a linear regression model was applied to illustrate the relationship between the observed and the performed movement mean velocities. The slopes of the model were considered as a percentage of contagion: 0 meant no modulation, while 1 referred to the perfect reproduction of the velocity of the observed action. Slope values were mainly used to compare the effects induced by motion observation on participants' responses, regardless of the differences in stimulus mean velocities. The statistical evaluation was obtained by means of a mixed-design ANOVA (Group as between-subject factor, Trajectory and Object-Directedness as within subject factors).

A repeated-measure ANOVA (with three levels of the factor Velocity) was performed on participants' mean movement velocities after the observation of the humanoid robot non-biological movement kinematics. The slopes of the regression lines obtained for each participant after non-biological and biological movement observation were statistically evaluated by means of a paired *t-test* to assess whether movement kinematics affected motor contagion mechanisms.

Significant interactions were always interpreted with Post-Hoc Newman-Keuls comparisons.

## Results

### Observers' movements were influenced by both human and robotic actions

**Figure 3:**
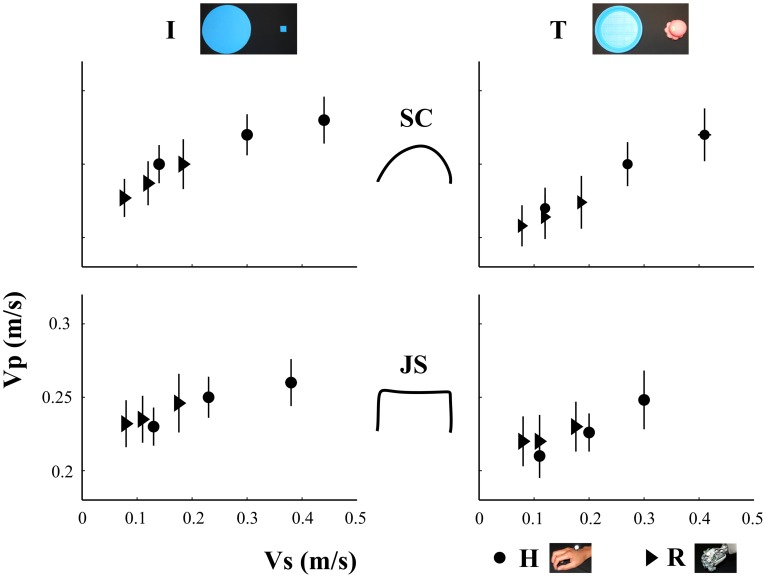
Participants' mean velocity (y-axis) as function of demonstrators' velocities (x-axis). Circles and triangles indicate mean V_P_ in response to the observation of human and humanoid demonstrators' movement, respectively. The columns –refer to intransitive (I-first) and transitive (T-second) movements. The first line displays the responses to smooth-curvilinear (SC) demonstrators' trajectories, while the second line to jerky-segmented (JS) stimulus trajectories. Vertical and horizontal error bars represent the participants and stimulus standard errors.

From a graphical inspection of Figure 3 it appears evident that participants' velocities varied consistently with stimulus velocities.

This was confirmed by the result of the statistical analysis on participants' mean movement velocities, which found a significant effect of the factor Velocity for both demonstrators and trajectories (see Table 3). Furthermore, a significant effect of Object-Directedness appeared when the robot moved along a smooth-curvilinear trajectory: i.e., participants' velocities were higher in the intransitive than in transitive condition. A significant interaction between Velocity and Object-Directedness was found only when observing the human demonstrator performing smooth trajectories. In particular, post hoc analysis showed that in the transitive condition the three movement velocities differed from each other (V_S_<V_M_<V_F_, *p<0.01*), whereas in the intransitive condition, V_S_ was found to be significantly lower than V_M_ and V_F_ (*p<0.01* in both cases).

**Table 3:** Results of the statistical analyses on the participants' mean velocity values (V_P_) in the different experimental conditions.

		Curvilinear trajectory	Segmented trajectory
**Humanoid robot**	Velocity	*F_2,26_ = 12.38, p<0.01*	*F_2,26_ = 3.96, p<0.05*
	Object-Directedness	*F_1,13_ = 7.53, p<0.05*	*Not significant*
	Velocity*Object-Directedness	*Not significant*	*Not significant*
**Human demonstrator**	Velocity	*F_2,24_ = 18.83, p<0.01*	*F_2,24_ = 14.84, p<0.01*
	Object-Directedness	*Not significant (p = 0.066)*	*Not significant (p = 0.055)*
	Velocity*Object-Directedness	*F_2,24_ = 8.08, p<0.01*	*Not significant*

To account for the differences in stimuli mean velocities a total of four repeated-measures ANOVAs were applied on V_P_ when observing the humanoid robot and the human demonstrator performing transitive and intransitive actions, while covering a smooth-curvilinear and jerky-segmented trajectories at different velocities.

Additionally, since the human demonstrator's velocity was found to be significantly higher when performing intransitive than transitive movements (*p<0.01*), to avoid any effect of this difference on participants' responses, we separately assessed the effect of Velocity in the transitive and intransitive conditions. Thus, we performed four additional ANOVAs (SC-T, SC-I, JS-T, JS-I) with Velocity as a unique factor. Results showed that participants' velocities varied coherently with stimulus velocities in each condition (always *p<0.01*).

**Figure 4:**
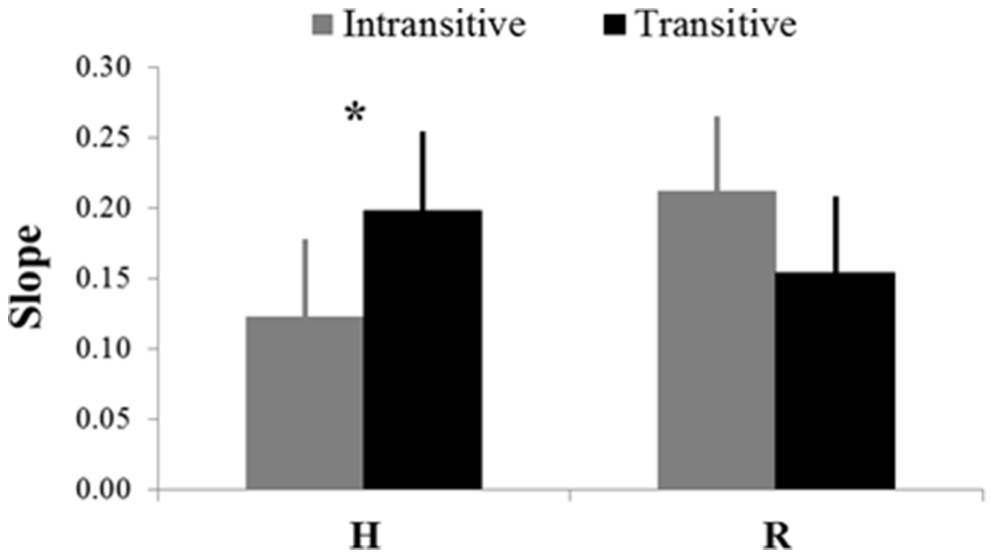
Slope values (mean±SE) of the regression lines that model the relationship between participants' and demonstrators' (human-H, robot-R) velocities. Grey and black columns refer respectively to intransitive and transitive conditions.

The statistical analysis on the slopes of the regression lines that modeled the relationship between demonstrator's and participants' V values in each experimental condition revealed only a significant Meaning*Group interaction (*F_1,25_ = 11.15, p<0.01*). In particular, post-hoc analysis showed that, when observing the human demonstrator, motor contagion increased significantly in transitive condition with respect to the intransitive task (*p<0.05*) (see Figure 4). Instead, no specific differences appeared between H and R for both Meaning conditions.

### Participants' movement velocity was not modulated by the observation of the humanoid robot when it moved with non-biological kinematics

**Figure 5:**
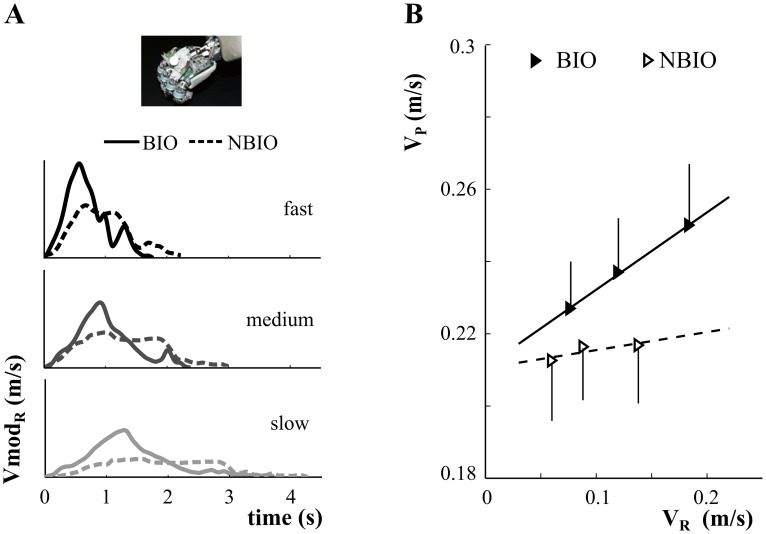
Robot's biological vs. non-biological kinematics. A) robot's biological (BIO, continuous line) and non-biological (NBIO, dashed line) velocity profiles are represented for the three movement velocities. While in BIO the deceleration phase started when the maximum of velocity was reached, in NBIO, after the initial acceleration phase, the velocity profile reached a maximum that was kept approximately constant for a minimum of 1 s, followed by a deceleration phase. B) Participants' mean velocity (V_P_, y-axis) as function of the robot's velocities (V_R_, x-axis) (mean±SE). Full and empty triangles refer to BIO and NBIO robot movement kinematics, respectively. Continuous and dashed traces are the regression lines which model V_P_ and V_R_ relationships in BIO and NBIO, respectively.

The role of the observed movement kinematics in speed contagion was evaluated. Since making the human demonstrator move according to a non-biological kinematics was not feasible, this analysis was performed only for the observation of the humanoid robot. Hence, the robot was programmed to move according to a biological (B) or non-biological (NB) law of motion (Figure 5A). Since the robot moved at different mean velocities in B and NB (Kinematics: *F(1,13) = 470.08, p<<0.01*), two separate one-way ANOVAs were employed to statistically evaluate participants' responses to B and NB kinematics observation.

When looking at biological motion, participants modulated their motor response coherently with stimulus velocity (Velocity: *F_2,26_ = 13.9, p<0.01*). In contrast, no differences appeared among participants' velocities when observing non-biological movement kinematics (Figure 5B).

The paired *t-test* used to compare the slopes of the regression lines that modelled the relationship among participants and robot mean velocities revealed a significant effect of the factor Kinematics (slope_NB_ = 0.06<0.24 = slope_B_: *t = 2.35, p<0.05*).

Therefore, altogether these data suggest that whereas after biological movement observation participants were influenced by robot movement velocity, in non-biological condition motor contagion disappeared.

## Discussion

This study aimed at understanding whether and how the nature of the visual stimulus and the properties of the observed actions influence the motor response of the observer. In particular, we focused on the comparison between properties with higher and lower cognitive value, namely the meaning and the kinematics, respectively. To investigate this issue, we applied a novel motor contagion paradigm in which participants were requested to look at a visual model, either human or humanoid, and to move their hands from one place to another, while handling or not handling an object, after the observation of the action of the demonstrator. The movement trajectory could be either smooth-curvilinear or jerky-segmented and was covered with a biological velocity profile. When the demonstrator was a robot we added a condition in which the smooth-curvilinear trajectory was performed also with a non-biological velocity profile. The results showed that the observed movement kinematics, namely an action property at low cognitive value, modulated the resonance mechanisms, whatever the nature of the observed stimulus, except when the humanoid robot violated the biological law of motion.

In case of biological kinematics, the same degree of motor contagion resulted from human and humanoid action observation. This finding was not easily predictable because the literature on human-robot interaction proposed contrasting results. In fact, both the early studies on mirror neurons system in the monkey [7], [8], and neuroimaging findings in humans [10] have cast doubts on the possibility for a humanoid agent to activate motor resonance. Our results are instead in line with more recent neuroimaging and neurophysiological experiments showing that the observation of a mechanical device induces motor resonance in humans (for reviews see [31], [32]). Moreover, we did not find a modulation in the resonating mechanism associated to humanoid action observation, confirming similar findings previously obtained in studies which applied different behavioral paradigms (i.e., [14], [33]–[40]). It is worth noting that the present work did not test the role of the robot appearance.

Although the non-human nature of the stimulus per se did not cancel motor resonance, a property of its movement – the velocity profile – was crucial to induce motor contagion. Indeed, when participants observed the demonstrators moving according to a biological law of motion, their movement velocity varied coherently with that of the stimulus. In contrast, when participants observed the robot performing movements with kinematics that where outside the human motor repertoire – i.e., violated the biological laws of motions – the stimulus velocity did not influence their responses. These results confirmed our previous findings [24], which showed that motor contagion appeared only when the observer was able to match his/her motor repertoire with that of the stylized visual model (i.e., a dot). Accordingly, motion inference was demonstrated to be dependent on the recognition of the observed motion repertoire [23]. A similar dominance of movement kinematics over the nature of the agent has been illustrated in a study by Grosjean et al. [41], in which the authors showed that Fitt's Law holds for action perception of both biological and non-biological agents. Thus, we propose that the biological kinematics of a moving stimulus played a predominant role to induce behavioral speed contagion with respect to other aspects of the observed motion. We speculate that this low-level property of the action is responsible for the activation of motor resonance mechanisms. Indeed, an action is stored in term of its kinematic and dynamic properties: thus, we suggest that the process of motor contagion originates from these components, namely from the observer's motor repertoire and not from the stimulus by itself. That is, motor contagion would appear only if the stimulus is compatible with the subjective motor properties.

The appearance of motor contagion when observing both smooth-curvilinear and jerky-segmented trajectories supports this interpretation. Indeed, even though unconventional, the “jerky-segmented” trajectory was feasible by a human agent, and thus it did not violate the biological law of motions. This idea is strengthened by a recent neuroimaging study showing that the observation of video depicting both human and robot performing either natural or rigid dancing movements induced activity in the action-observation network (i.e. parietal, premotor and occipito-temporal regions) [27]. Furthermore, these findings are in line with a series of neurophysiological studies focused on evaluating the contribution of different dimensions of action, such as the kinematics and the goal, indicating that the observed action matched the observer motor system at low (i.e., kinematics) level [20]–[22]. Thus, our findings suggest that low-level representation of movement is crucial to evoke motor contagion while other properties, such as the congruence between the nature of the visual stimulus and the shape of its motion, do not affect motor resonance mechanisms. Furthermore, it is worth noting that this work, for the first time, successfully dissociated the consequences of the observation of two aspects of movement kinematics that are usually not disentangled, namely the path and the velocity profile, by showing how they differently affected motor contagion processes.

In conclusion, the present results suggest that motor resonance is a very robust mechanism, not limited by the nature of the visual model and by the external appearance of its motion. On the contrary, its occurrence seems to rely on the possibility for the observer to match her/his motor repertoire with that of the model. This idea is in line with the direct matching hypothesis stating that motor resonance might be based on a mechanism directly matching the observed action onto an internal motor representation of that action [3].

Interestingly, however, when looking at the human agent, the influence of the stimulus increased for transitive relative to intransitive movement observation. Notably, in the intransitive condition participants just moved the hand from one place to another, while in the transitive condition the action she/he observed and executed had an explicit meaning, namely to put the octopus in its container. Thus, the goal of the action seems to affect the contamination process. One can hypothesize that when facing a moving agent the observer aims to understand his future action. Although the recognition of his movement kinematics could help to predict action evolution [3], [23], other contextual static visual input, such as the presence of an object, can feed the “intention tracking” mechanism, boosting the resonance process. No such increase of motor contagion for transitive movement observation occurred when the humanoid robot performed actions. We speculate that this effect could depend on a less refined intention-attribution mechanism, which in case of a robotic agent, does not distinguish between low level (or location) goals and more concrete, object-related goals. Indeed, the fact that the observed motion may exert different effects on observer's motor response might depend on the perceived intentionality of the agent's gesture, as suggested by Sartori and colleagues [15]. A growing body of literature investigated cues for triggering intention attribution [42], [43] and factors, such as goal-directedness actions [44] and gaze direction [14], [45] have been invoked as responsible for intention attribution. Nevertheless, in order to provide clearer explanation, this result needs to be specifically addressed in future works.

### Conclusions

In this work we showed that motor resonance mechanisms, in the form of motor contagion, can appear when observing both human and non-human actions. Although the shape of the trajectory did not affect these processes, the observation of non-biological velocity profile prevented the observer's motor system from resonating with that of the model. Thus, we can conclude that the possibility for the observer to match his/her own motor repertoire with that of the observed stimulus might mediate the activation of motor resonance and, consequently, modulate the spontaneity and the pleasantness of the interaction, whatever the nature of the communication partner.

Together with the characterization of the behavioral consequences of motor resonance mechanism during human-human interaction, this work offers an insight into the way humans perceive and react to non-human agents. Indeed, the present findings shed light on the kind of interaction humans can establish with humanoid robots [31], [32], a new kind of social agents expected to co-exist with humans, sharing the same working space and assisting them during daily life activities. Indeed, since motor resonance was proposed to underlie spontaneous and pleasant relations [4], [46]–[48], the activation of these mechanisms during human-robot interaction would guarantee natural and human-like communication also with non-human beings. In summary, in light of a scenario in which humans will co-exist and cooperate with humanoid robots, the possibility to consider robots as a social, interaction partners and to establish a natural relationship with them seems to be inevitable.
